# Data on molecular characterization and expression profiles of the NF-κB repressing factor gene in red sea bream (*Pagrus major*)

**DOI:** 10.1016/j.dib.2019.103977

**Published:** 2019-05-22

**Authors:** Kwang-Min Choi, Jee Youn Hwang, Mun-Gyeong Kwon, Ji-Min Jeong, Jung Soo Seo, Seong Don Hwang, Bo-Yeong Jee, Mu-Chan Kim, Chan-Il Park

**Affiliations:** a Institute of Marine Industry, College of Marine Science, Gyeongsang National University, 455, Tongyeong 650-160, Republic of Korea; b Aquatic Animal Disease Control Center, National Institute of Fisheries Science (NIFS), 216 Gijanghaean-ro, Gijang-eup, Gijang-gun, Busan 46083, Republic of Korea

**Keywords:** NF-κB repressing factor, *Pagrus major*, *Streptococcus iniae*, Red sea bream iridovirus

## Abstract

Nuclear factor-kappaB (NF-κB) repressing factor (NKRF) specifically inhibits the transcriptional activity of NF-κB protein. The PmNKRF cDNA is composed of 757 amino acid residues. Alignment analysis revealed that the G-patch and R3H domains are conserved in different organisms. We aimed to analyse red sea bream NKRF (PmNKRF) gene expression after infection with pathogens [*Streptococcus iniae* or red sea bream iridovirus (RSIV)] and in healthy individuals. In healthy individuals, PmNKRF was ubiquitously expressed in all 12 tested tissues, predominantly in the head kidney and spleen. Expression of PmNKRF was significantly up-regulated in the gills, kidney, liver and spleen after RSIV infection. After *S. iniae* infection, PmNKRF expression was significantly down-regulated in the gills and significantly up-regulated in the kidney, liver and spleen.

**Table:** Specifications table

Subject area	*Immunology and Microbiology*
More speciﬁc subject area	*Immunology*
Type of data	*Table and figure*
How data was acquired	*GENETYX version 8.0, MEGA version 6.0 and Real-Time PCR*
Data format	*Raw and Analysed*
Experimental factors	*PmNKRF gene expression profiles were compared between healthy controls and groups with bacterial and viral infections.*
Experimental features	*The sequence of the PmNKRF gene was identified by NGS analysis, and its molecular and expression characteristics were confirmed.*
Data source location	*Gyeongsang National University, Tongyeong, Republic of Korea*
Data accessibility Related research article	*The data are available with this article*
	*M. Nourbakhsh, H. Hauser, Constitutive silencing of IFN-beta promoter is mediated by NRF (NF-kappaB-repressing factor), a nuclear inhibitor of NF-kappaB, EMBO J., 18 (1999), pp. 6415-6425*

**Table:** 

**Value of the data** • These data confirmed the possibility of mRNA expression characteristics of PmNKRF, which is important for the transcription of pathogenic and cytokine-stimulated genes in the red sea bream immune system. • The present data will be fundamental to improving the understanding of the role of PmNKRF in the immune response of the red sea bream due to pathogenic infections. • These data will help understand the role of nuclear factor-kappaB pathway in the immune system of teleosts. • Based on these tissue-specific expression data sets, further studies on related genes will contribute to gaining insights into the limited functions of NKRF in teleosts.

## 1 Data

**Fig. 1:**
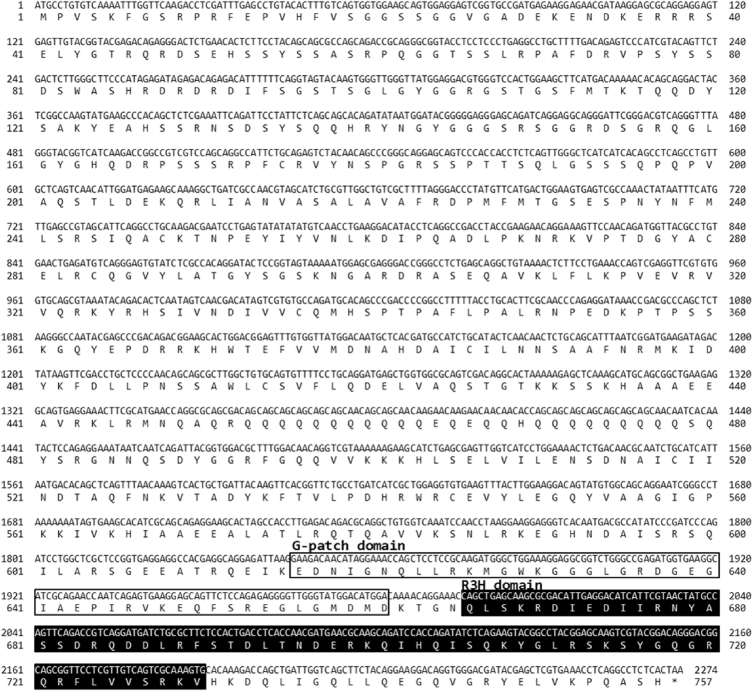
Nucleotide and deduced amino acid sequences of PmNKRF. The box and black box indicates a G-patch domain and R3H domain, respectively.

**Fig. 2:**
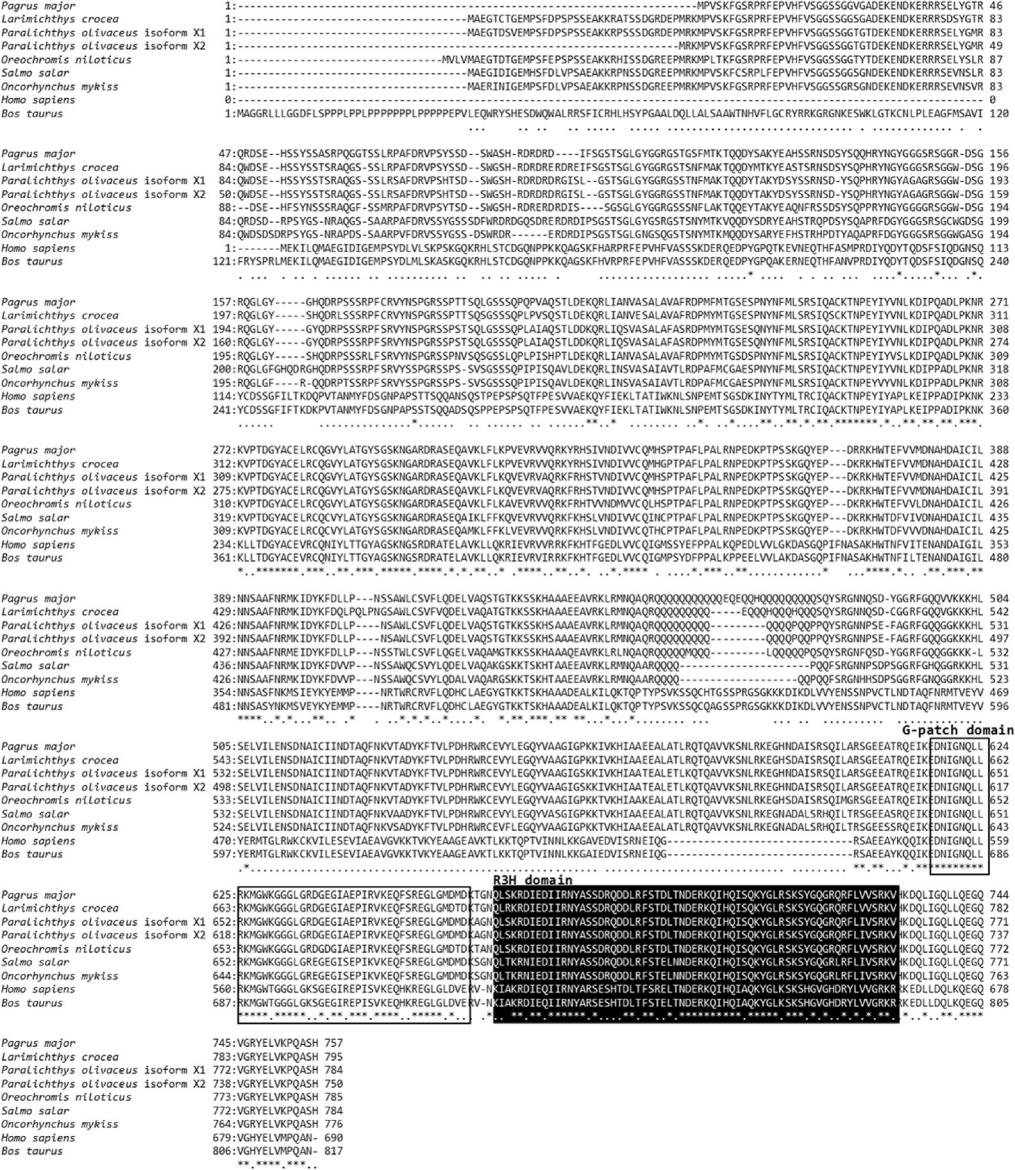
Comparisons of the PmNKRF amino acid sequence with other known NKRF sequences of species. Amino acids that are identical to the red sea bream (*Pagrus major*) sequence are indicated by asterisks (*), similar amino acid residues are indicated by dots (.), and the G-patch domain and R3H domain are indicated by the box and black box, respectively.

**Fig. 3:**
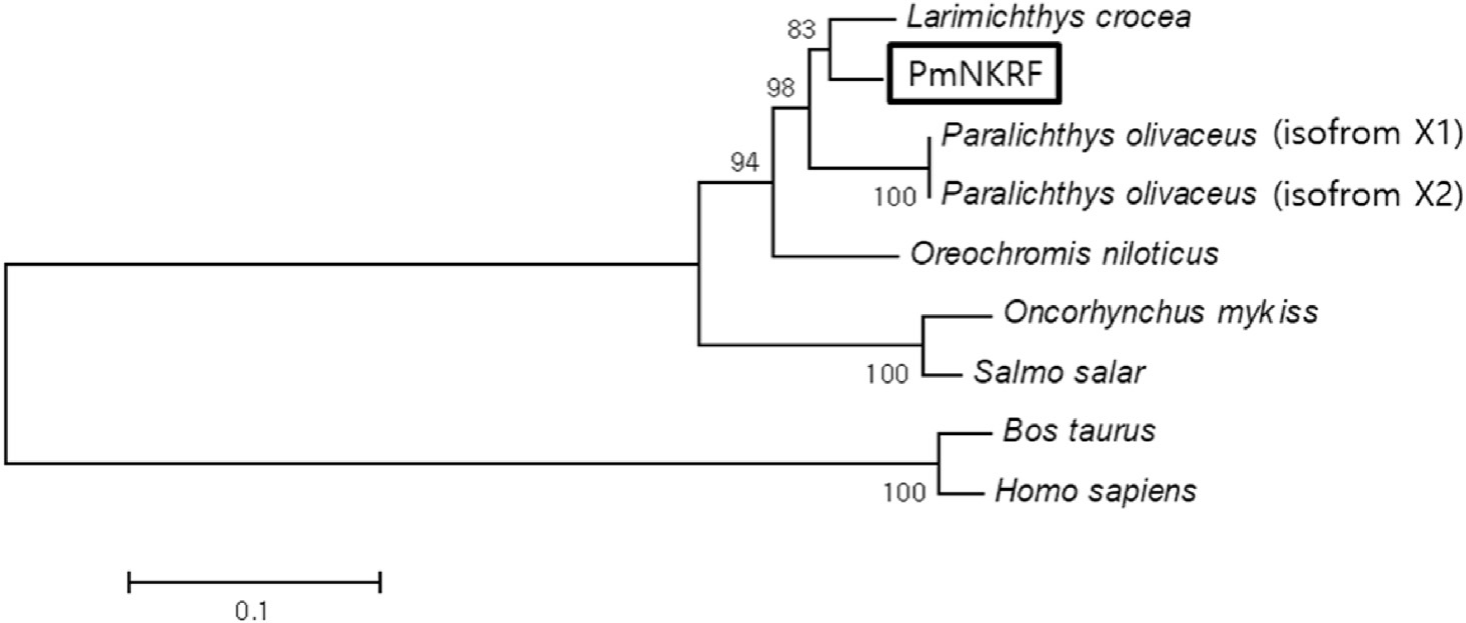
Phylogenetic tree based on amino acid sequence of NKRF downloaded from GenBank. The phylogenetic tree was constructed by the neighbor-joining method. Bootstrap values shown at the nodes of the tree are based on 2000 bootstrap replicates. The scale bar represents sequence divergence. GenBank accession number: *Larimichthys crocea* (XP_019116279); *Paralichthys olivaceus* isoform X1 (XP_019937504); *Paralichthys olivaceus* isoform X2 (XP_019937513); *Oreochromis niloticus* (XP_005467451); *Salmo salar* (NP_001158819); *Oncorhynchus mykiss* (XP_021417162); *Homo sapiens* (AAH68514); *Bos taurus* (XP_005227485).

Nuclear factor-kappaB (NF-κB) repressing factor (NKRF) has been reported to specifically inhibits the transcriptional activity of NF-κB protein [1], [2]. An open reading frame (ORF) containing the NKRF sequence of red sea bream (PmNKRF) was identified by next generation sequencing (NGS) analysis of liver samples from bacteria infected red sea bream (GenBank accession number: AXG64253). The PmNKRF cDNA contained an ORF of 2274 bp encoded a 757 amino acids. The G-patch and R3H domains were identified in the amino acid sequence of PmNKRF both domains were conserved at a high level in multiple alignment analysis with NKRF gene of other species (Fig. 1, Fig. 2). Phylogenetic analysis showed that PmNKRF was included in the cluster of marine fishes and showed the closest affinity to NKRF of *Larimichthys crocea*, with a homology of 92.7% (Fig. 3).

**Fig. 4:**
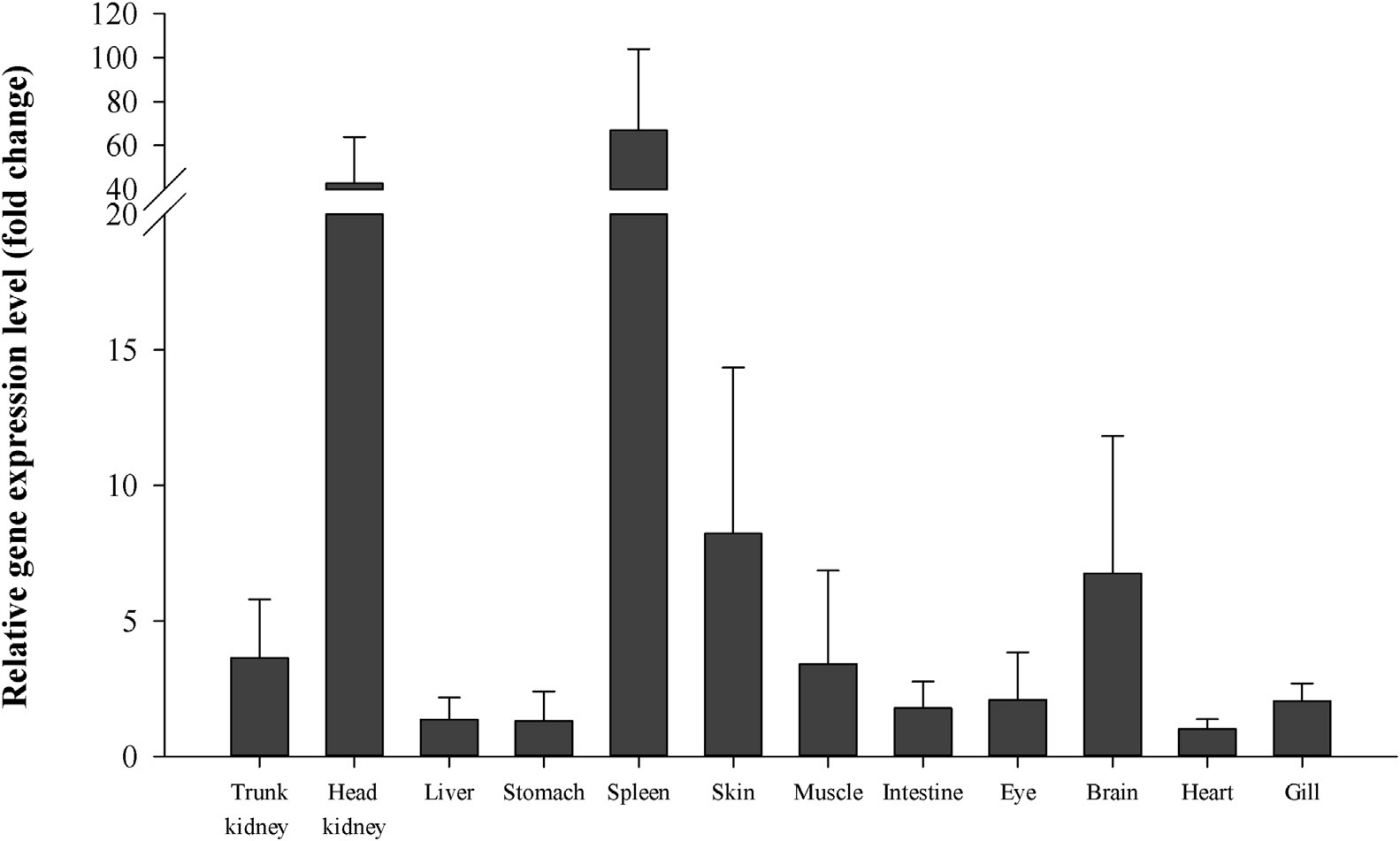
Gene expression analysis of PmNKRF gene by RT-qPCR in various tissues of healthy red sea bream. The EF-1α gene was used to normalise the RT-qPCR data. The experiments were repeated three times and data are presented as the means ± SD versus the control (heart).

**Fig. 5:**
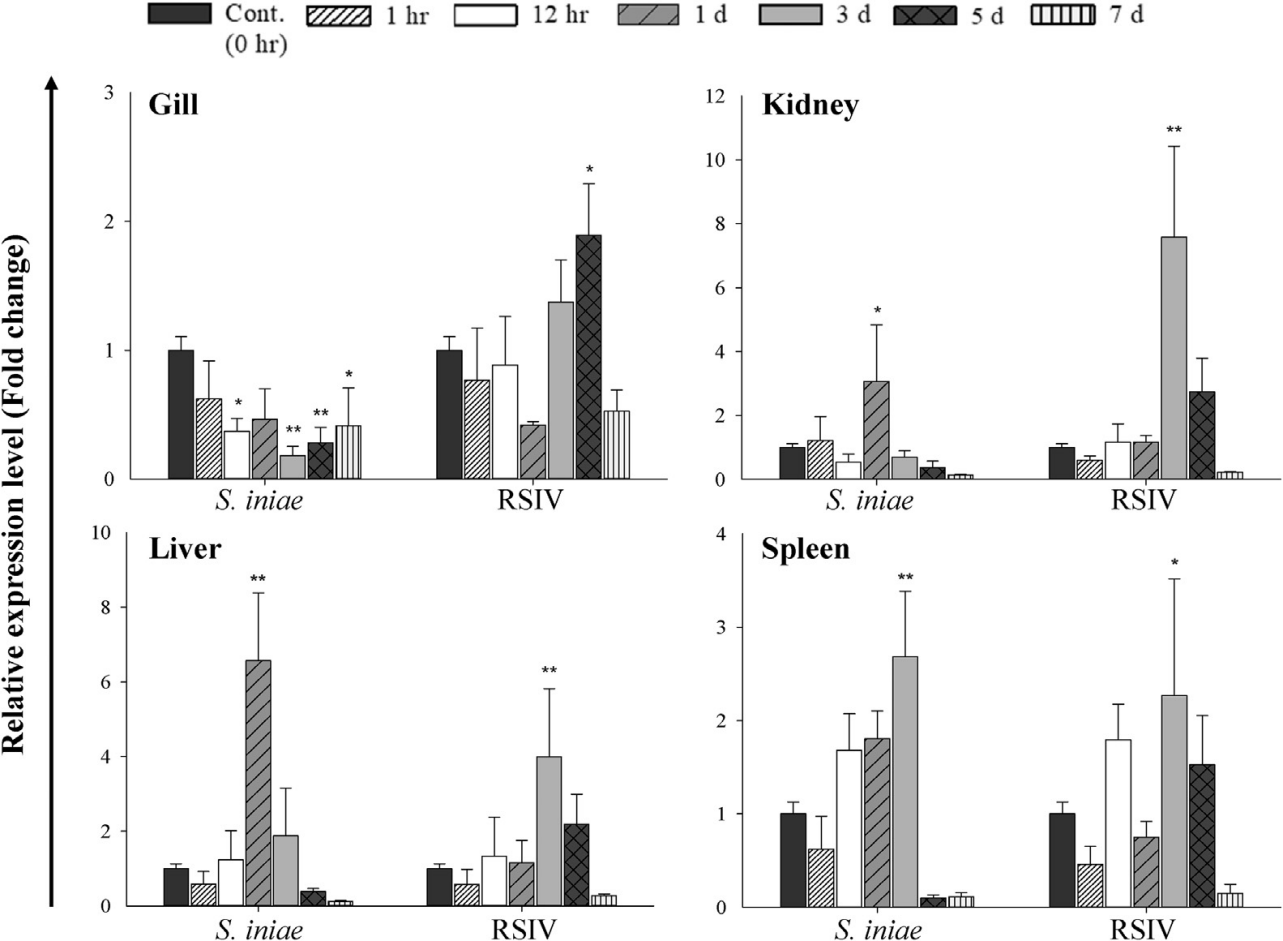
Expression analysis of PmNKRF mRNA in kidney, spleen, gill and liver of red sea bream after infection with *S. iniae* or RSIV using RT-qPCR. PmNKRF was quantified relative to that of the EF-1α gene. Gene expression and its significance are represented as mean ± SD (N = 5). Asterisks indicate significant differences (*P < 0.05, **P < 0.01) versus the control (0 h).

Using quantitative real-time PCR (RT-qPCR), we evaluate NKRF mRNA expression in healthy and pathogen challenged red sea bream (*Pagrus major*). In healthy red sea bream, NKRF (PmNKRF) mRNA displayed significantly higher expression levels in the spleen (67.1 fold) and head kidney (42.7 fold) compared to the heart (Fig. 4, Table 1). In analysing gene expression after pathogen challenge, PmNKRF expression was significantly up-regulated in the kidney, liver and spleen at 1 days, 1 days and 3 days, respectively, after *Streptococcus iniae* (*S. iniae*) infection, and significantly down-regulated in the gills (Fig. 5, Table 2). During red sea bream iridovirus (RSIV) infection, PmNKRF mRNA was significantly up-regulated at later timepoints (3 or 5 days) and then decreased in all tissues used in the experiment.

**Table 1:** Expression level of PmNKRF mRNA in various tissues of healthy red sea bream.

Tissue	ΔΔCt value	Tissue	ΔΔCt value
Trunk kidney	8.08	Muscle	9.13
	6.82		8.74
	8.28		6.55
Head kidney	5.01	Intestine	7.95
	3.44		9.69
	4.01		8.67
Liver	8.24	Eye	8.78
	9.45		7.42
	9.73		10.06
Stomach	8.07	Brain	5.78
	9.96		7.37
	10.09		7.64
Spleen	3.68	Heart	9.11
	4.18		10.24
	2.67		9.20
Skin	9.25	Gill	8.69
	5.93		8.70
	5.83		7.95

**Table 2:** Expression level of PmNKRF mRNA in various tissues after pathogen challenge.

Tissue	Time points	ΔΔCt value				ΔΔCt value	
		S. Iniae	RSIV			S. iniae	RSIV
	0 hours	7.21	7.21		0 hours	8.04	8.04
		7.09	7.09			7.92	7.92
		6.91	6.91			7.72	7.72
	1 hour	8.84	6.9		1 hour	6.85	8.83
		7.54	7.29			7.91	8.86
		7.29	8.68			8.65	8.31
	12 hours	8.21	6.95		12 hours	9.94	7.62
		9.02	8.21			8.42	8.68
		8.38	6.9			8.45	7.11
Gill	1 day	7.51	8.29	Kidney	1 day	5.74	7.68
		8.84	8.24			6	7.91
		8.51	8.42			7.78	7.43
	3 days	9.65	6.6		3 days	8.49	5.49
		10.12	7			8.86	5.14
		9.01	6.3			8.02	4.46
	5 days	8.69	5.9		5 days	8.85	5.18
		8.48	6.52			10.74	7.61
		9.83	6.08			9.04	8.97
	7 days	9.49	8.62		7 days	11.06	10.09
		8.77	7.68			10.52	9.9
		7.49	7.83			10.84	10.11
	0 hours	7.47	7.47		0 hours	5.89	5.89
		7.81	7.81			6.18	6.18
		7.49	7.49			6.21	6.21
	1 hour	7.62	10.38		1 hour	6.07	7.77
		8.98	8.24			7.01	7.43
		8.9	7.66			7.71	6.65
	12 hours	6.71	7.26		12 hours	5.18	6.65
		9.12	9.18			5.13	4.98
		6.98	6.31			5.79	5.21
Liver	1 day	5.18	6.85	Spleen	1 day	5.03	6.32
		4.47	8.58			5.2	6.93
		5.05	7.19			5.51	6.33
	3 days	8.8	5.62		3 days	5.13	4.58
		6.33	6.44			4.35	6.34
		6.08	5.03			4.61	4.44
	5 days	9.29	5.98		5 days	10.06	5.24
		8.92	6.52			9	6.21
		8.69	7.05			9.41	5.19
	7 days	10.54	9.67		7 days	10.11	8.95
		10.92	9.39			8.77	10.2
		10.4	9.24			9.25	8.09

## 2 Experimental design, materials, and methods

### 2.1 Molecular characterisation and phylogenetic analysis

An ORF containing the sequence of PmNKRF was identified from the liver samples from bacteria infected red sea bream by NGS analysis. Sanger sequencing was performed to confirm the integrity of the cDNA sequence. The nucleotide sequences and predicted amino acid sequences of PmNKRF were analyzed by the BLAST algorithm of the National Centre for Biotechnology Information (http://www.ncbi.nlm.nih.gov/blast). Species and the corresponding GenBank accession numbers are as follows: *Larimichthys crocea* (XP_019116279); *Paralichthys olivaceus* isoform X1 (XP_019937504); *Paralichthys olivaceus* isoform X2 (XP_019937513); *Oreochromis niloticus* (XP_005467451); *Salmo salar* (NP_001158819); *Oncorhynchus mykiss* (XP_021417162); *Homo sapiens* (AAH68514); *Bos taurus* (XP_005227485). Based on amino acid sequences, the characteristic domain was predicted using the Expert Protein Analysis System PROSITE Scan tool (http://prosite.expasy.org). Multiple sequence alignment was performed with the GENETYX software version 8.0 (SDC Software Development, Japan). A phylogenetic tree was constructed with Molecular Evolutionary Genetics Analysis (MEGA) version 6.0 using the neighbour-joining method. The primer sets of PmNKRF and PmEF-1α (the reference gene) used in the experiments were designed using primer3 program.

### 2.2 Experimental animal and microbes

Healthy red sea bream (weight: 173.2 ± 31.1 g, body length: 22.4 ± 0.9 cm) were obtained from the Gyeongsangnam-do Fisheries Resources Research Institute (Tongyeong, Republic of Korea), maintained at 22 ± 1 °C in aerated seawater and fed daily with commercial dry pellets. In all experiments, animals were euthanized by anesthesia before tissue collection.

The microbial strains used in this data, *S. iniae* and RSIV were provided by the Fish Pathology Division of the National Institute of Fisheries Science (Busan, Republic of Korea). Bacteria were cultured in brain heart infusion medium at 27 °C.

### 2.3 RT-qPCR analysis of PmNKRF in different tissues

Relative PmNKRF mRNA levels in various tissues were determined by RT-qPCR. Total RNA was extracted from various tissues (trunk kidney, head kidney, liver, stomach, spleen, skin, muscle, intestine, eye, brain, heart and gill) of three healthy red sea bream using a TRIzol-based (RNAiso Plus) reagent (Takara, Japan) according to the manufacturer's instructions. The total RNA samples were subjected to DNase I (Takara) treatment to remove contaminating genomic DNA. The concentration and purity of the total RNA samples were calculated from measurements obtained by a NanoVue spectrophotometer (GE Healthcare, UK). Total RNA was used for cDNA synthesis using a 1st strand cDNA synthesis kit (Takara) according to the manufacturer's instructions. Finally, RT-qPCR was performed with a gene-speciﬁc primer set (Forward: 5′-CACCTCTCAGTTGGGCTCAT-3′ and Reverse: 5′-GGCGACTCACTTCCAGTCAT-3′) on a Thermal Cycler DICE Real-Time System (Takara) using TB Green™ Premix Ex Taq™ (Takara). The relative expression level of PmNKRF was calculated using the comparative threshold cycle method (2^−ΔΔCT^) with elongation factor-1α used as a control (Forward: 5′-CCTTCAAGTACGCCTGGGTG-3′ and Reverse: 5′-CTGTGTCCAGGGGCATCAAT-3′). The data sets are expressed as the relative fold change normalized to that of heart tissue.

### 2.4 RT-qPCR analysis of PmNKRF after pathogen infection

Infection experiments were performed by intraperitoneal injection of a pathogen suspension of *S. iniae* (10^5^ CFU/fish) or RSIV (10^6^ copies/fish). Three fish in each time were collected for RT-qPCR at 0, 1 and 12 hours and 1, 3, 5 and 7 days post-infection, and the kidney, gills, liver and spleen were harvested. The mRNA expression of PmNKRF in the tissue of infected red sea bream was measured by RT-qPCR as above.

Data were assessed using a one-way analysis of variance (ANOVA) followed by Tukey's test (*P value < 0.05 and **P value < 0.01) using the SPSS software 19.0 (IBM, USA). All samples were analysed in triplicate; the data are reported as the mean ± standard deviation (SD).
