# Supplementary material for: Data on molecular characterization and expression profiles of the NF-κB repressing factor gene in red sea bream (Pagrus major)
Source: Data Brief. 2019 May 22;24:103977. doi: 10.1016/j.dib.2019.103977 (PMC6545398; doi:10.1016/j.dib.2019.103977)
Supplement: Multimedia component 1 [file mmc1.pdf]

Conflict of interest declaration and author agreement form

It is important that you return this form upon submission. We will not publish your article without completion and return of this form.

Title of Paper: Data on molecular characterization and expression profiles of the NF-κB repressing factor gene in red sea bream (*Pagrus major*)

Please tick one of the following boxes:

☑ We have no conflict of interest to declare.

☑ We have a competing interest to declare (please describe below):

This statement is to certify that all Authors have seen and approved the manuscript being submitted. We warrant that the article is the Authors' original work. We warrant that the article has not received prior publication and is not under consideration for publication elsewhere. On behalf of all Co-Authors, the corresponding Author shall bear full responsibility for the submission.
This research has not been submitted for publication nor has it been published in whole or in part elsewhere. We attest to the fact that all Authors listed on the title page have contributed significantly to the work, have read the manuscript, attest to the validity and legitimacy of the data and its interpretation, and agree to its submission to Data in Brief.
All authors agree that author list is correct in its content and order and that no modification to the author list can be made without the written acceptance of all authors and the formal approval of the Editor-in-Chief. All authors accept that the Editor-in-Chief's decisions over acceptance or rejection or in the event of any breach of the Principles of Ethical Publishing in Data in Brief being discovered, of retraction are final.
Upon acceptance, the Author assigns to the Data in Brief the right to publish and distribute the manuscript in part or in its entirety. The Author's name will always be included with the publication of the manuscript.
The Author has the following nonexclusive rights: (1) to use the manuscript in the Author's teaching activities; (2) to publish the manuscript, or permit its publication, as part of any book the Author may write; (3) to include the manuscript in the Author's own personal or departmental (but not institutional) database or on-line site; and (4) to license reprints of the manuscript to third persons for educational photocopying. The Author also agrees to properly credit the Data in Brief as the original place of publication.

Print Name Author Signature Date

□ Please check this box if you are submitting this on behalf of all authors.

This statement is signed by all the authors to indicate agreement that the above information is true and correct

Author's name	Author's signature	Date
Kwang-Min Choi		April 18, 2019
Jee Youn Hwang		April 18, 2019
Mun-Gyeong Kwon		April 18, 2019
Jung Soo Seo		April 18, 2019
Seong Don Hwang		April 18, 2019
Bo-Yeong Jee		April 18, 2019
Mu-Chan Kim		April 18, 2019
Chan-Il Park		April 18, 2019
